# GAMETOPHYTE DEFECTIVE 1, a Putative Subunit of RNases P/MRP, Is Essential for Female Gametogenesis and Male Competence in Arabidopsis

**DOI:** 10.1371/journal.pone.0033595

**Published:** 2012-04-11

**Authors:** Si-Qi Wang, Dong-Qiao Shi, Yan-Ping Long, Jie Liu, Wei-Cai Yang

**Affiliations:** State Key Laboratory of Molecular Developmental Biology, National Centre for Plant Gene Research, Institute of Genetics and Developmental Biology, Chinese Academy of Sciences, Beijing, China; Temasek Life Sciences Laboratory, Singapore

## Abstract

RNA biogenesis, including biosynthesis and maturation of rRNA, tRNA and mRNA, is a fundamental process that is critical for cell growth, division and differentiation. Previous studies showed that mutations in components involved in RNA biogenesis resulted in abnormalities in gametophyte and leaf development in *Arabidopsis*. In eukaryotes, RNases P/MRP (RNase mitochondrial RNA processing) are important ribonucleases that are responsible for processing of tRNA, and transcription of small non-coding RNAs. Here we report that *Gametophyte Defective 1* (*GAF1*), a gene encoding a predicted protein subunit of RNases P/MRP, AtRPP30, plays a role in female gametophyte development and male competence. Embryo sacs were arrested at stages ranging from FG1 to FG7 in *gaf1* mutant, suggesting that the progression of the gametophytic division during female gametogenesis was impaired in *gaf1* mutant. In contrast, pollen development was not affected in *gaf1*. However, the fitness of the mutant pollen tube was weaker than that of the wild-type, leading to reduced transmission through the male gametes. GAF1 is featured as a typical RPP30 domain protein and interacts physically with AtPOP5, a homologue of RNases P/MRP subunit POP5 of yeast. Together, our data suggest that components of the RNases P/MRP family, such as RPP30, play important roles in gametophyte development and function in plants.

## Introduction

RNA biogenesis is an essential biochemical process that not only plays a housekeeping role for cellular life, but also mediates many aspects of vegetative and reproductive development. Studies on genes encoding essential proteins in RNA biogenesis have attracted more and more attention since last decades, and many reports have argued that ribosomal proteins or components for ribosome biogenesis, are involved in the cellular process, organ and organism development [1], [2], [3]. Various studies also indicated that mutations in genes involved in these processes result in fertility failure in *Arabidopsis* [4], [5]. However, most of the reports have shown that genes involved in RNA processing often affect female gametophyte development more severely than that of the male. Consistently, expression profiling of female gametophytic cells reveals that genes encoding PAZ, PIWI domain or DUF1785 protein family involved in tRNA, rRNA, or mRNA processing, also exhibit high level of expression in the embryo sac [6]. The mutations in *SLOW WALKER* genes, such as *SWA1*, *SWA2* and *SWA3*/*AtRH36*, impair the mitotic cell cycle progression during female gametogenesis, causing female sterility in *Arabidopsis* [7], [8], [9], [10]. *SWA1* is homologous to the yeast UTP15 and plays a role in 18S pre-rRNA processing [7]. SWA2 is most likely involved in export of pre-ribosomes from nucleus to cytoplasm [8]. SWA3, or AtRH36, encoding a putative RNA helicase, is also required for 18S pre-rRNA processing [9], [10]. Similarly, *NUCLEOLAR FACTOR 1* (*NOF1*), which is involved in nucleolar functions and rRNA expression, is required for embryogenesis and female gametogenesis [11]. Besides the mitotic progression during embryo sac development, genes involved in RNA biogenesis are also reported to play a role in gametophytic cell fate, polar nuclei fusion, and female control of pollen tube attraction. For example, *MAGATAMA3* (*MAA3*), a gene involved in many aspects of RNA metabolism, is essential for the polar nuclei fusion and pollen tube guidance [12]. These data suggest that the regulation of rRNA processing and ribosome biogenesis is essential for female gametophyte development and function. In addition, mutations in genes coding for proteins involved in pre-mRNA splicing, such as *LACHESIS* (*LIS*), *GAMETOPHYTIC FACTOR1* (*GFA1*)/*CLOTHO* (*CLO*) and *ATROPUS* (*ATO*), also disrupt female gametophyte development. Intriguingly, these genes seem to play a yet unknown role in cell fate specification of the embryo sac [4], [5], [6], [13], [14], [15], [16], [17]. In *lis* mutant, the synergid and central cell adopted egg cell fate, leading to supernumerary egg cells [14]. *gfa1* embryo sacs produce incorrect number of nuclei, aberrant cellular structure, and delayed maturation, as well as defects in embryo and pollen development [9], [13]. Mutation in *ATO* also causes abnormal specification of the gametic cell fate [15].

As one of the most important RNA metabolism components, Ribonuclease P (RNase P) has been under extensive research since its discovery. RNases P and MRP are essential site-specific endoribonucleases that are involved in processing the 5′end of pre-tRNAs. RNase MRP was evolved from nuclear RNase P, and is responsible for pre-rRNA processing [18], [19], [20]. They share most of the protein subunits and structural features of their RNA subunits [21], [22], [23], [24], [25], [26]. The structure and sequence of the RNA and protein components are highly conserved in eukaryotic kingdom [27]. Besides some common components, they also have specific RNAs and proteins individually. Recently, additional roles of eukaryotic RNases P/MRP have been revealed. It was shown that RNase P may play a role in stress response [28] and transcription by regulating RNA polymerase III as a transcription factor [29], [30]. In yeasts, RNase MRP is involved in cell cycle regulation by cleaving *CLB2* (cyclin B2) mRNA [27], [31], [32]. Loss of function of these two ribonucleases would cause serious consequences. In yeast, for example, *rpp1* and *rpp2* mutants are inviable [33], [34]. Reduced expression of the RNA component of MRP causes the multifaceted human Cartilage-Hair Hypoplasia (CHH) disease [35], [36], Pin Schmid metaphyseal chondrodysplasia (MDWH) disease [37], [38], or anauxetic dysplasia disease [39]. These suggest a unique role of RNases P/MRP in both yeast and animal cells.

In plants, however, little is known about RNases P/MRP. Two MRP RNAs have been found in *Arabidopsis* and one in rice, respectively. However, no P RNA has been discovered in RNase P, which implies that P RNA genes in plants may be very different with the known genes in the nuclear genome [40]. Nevertheless, the function and composition of RNases P and MRP remain to be elucidated in plants. Here we report the detailed genetic and molecular characterization of a mutant, *gametophyte defective 1* (*gaf1*), in which a gene encoding a predicted *Arabidopsis* RNases P/MRP protein subunit RPP30 is disrupted. Female gametophytic development and male competence were compromised in *gaf1*. Furthermore, we also showed that GAF1 could interact with another predicted *Arabidopsis* RNases P/MRP protein subunit AtPOP5 in yeast cells. All these data indicated that GAF1 is a subunit of RNases P/MRP that plays a role in gametophyte development and function in plants.

## Results

### Isolation of gametophytic mutants

**Figure 1:**
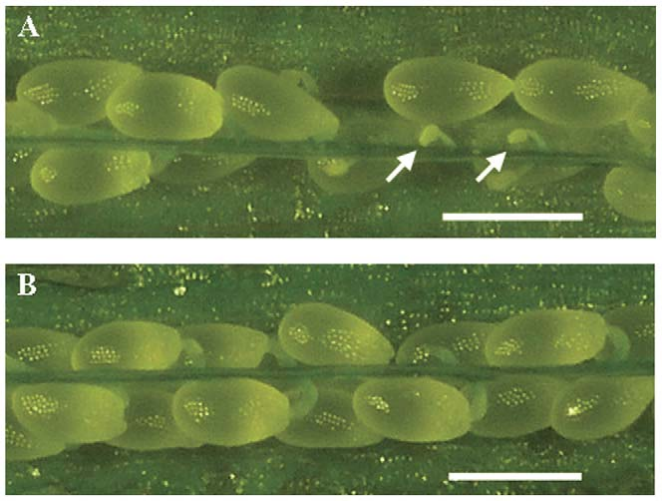
Reduced seed set in *gaf1* mutant silique. A. A *gaf1* silique showing aborted ovules (white arrows). B. A wild-type silique with full seed set. Bar=500 µm.

Gametogenesis is a fine-tuned developmental process. During female gametogenesis, one of the meiotic products–the functional megaspore–undergoes three rounds of mitosis that generates an eight-nucleate coenocyte, subsequently, two polar nuclei migrate and fuse, thereafter simultaneous cellularization of the coenocyte occurs to produce the mature seven-celled female gametophyte [41], [42], [43]. During male gametogenesis, microspore undergoes two consecutive rounds of mitosis, namely PMI (pollen mitosis I) and PMII, to generate a tricellular male gametophyte, the pollen [44], [45], [46]. To dissect the function of RNases P/MRP in gametogenesis and plant development, gametophytic mutants showing segregation distortion and reduced seed set were isolated from the *Ds* insertion pool and their target genes were identified [7], [47]. *gaf1* mutant in which a gene encoding a putative RNases P/MRP component, was identified. Molecular analysis showed that a single copy of *Ds* element was present in *gaf1*. Instead of the typical 3∶1 segregation ratio for sporophytic recessive mutation, self-pollinated *gaf1* progenies showed a 0.63∶1 (213∶338) segregation ratio of kanamycin resistant (Kan^R^) to kanamycin sensitive (Kan^S^), which is typical for mutants defective in both male and female gametophytes. Consistently, the siliques of heterozygous *gaf1* mutant contained 48.5% (324 in 668 ovules) aborted ovules (Figure 1). 

In order to further confirm the effects of the mutation on both male and female gametophytes, reciprocal crosses between heterozygous *gaf1* and the wild-type plants were conducted. When *gaf1* was used as the pollen donor, about 34.4% of the F1 progeny (n=1201) were Kan^R^, indicating that the male gametophyte development or function was defective. When *gaf1* was used as the egg donor, only about 0.46% of the F1 progeny (n=658) showed Kan^R^, suggesting that the female gametophyte development or function was severely impaired (Table 1). Consistently, no homozygote was recovered. We use *gaf1* to represent *gaf1/GAF1* heterozygous mutant in this report. Taken together, *gaf1* is a gametophytic mutation which affects both male and female gametophytes with a stronger effect on the female than the male.

**Table 1:** Results of reciprocal and self-crosses of *gaf1/GAF1* mutant.

Transmission	Cross	Kan^R^	Kan^S^	Kan^R^(expected)	TE
Male	+/+ X *Ds*/+	413	788	34.4% (50%)	52.4%
Female	*Ds*/+ X +/+	3	655	0.46% (50%)	0.46%
Self	*Ds*/+ X *Ds*/+	213	338	38.7% (75%)	

### The mutation impairs developmental progression of the female gametophyte

**Figure 2:**
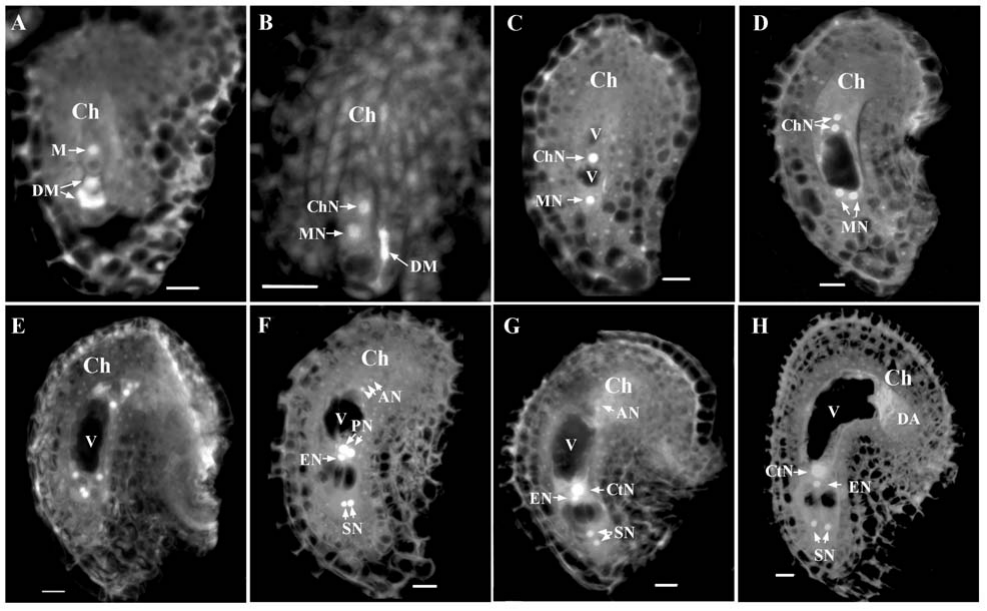
Development of wild-type female gametophyte. A. A female gametophyte at FG1 showing functional megaspore (M) and degenerated megaspores (DM). B. A female gametophyte at FG2 with one nucleus at the chalazal pole (ChN), and the other nucleus at the micropylar pole (MN). The degenerated megaspores (DM) still present. C. A female gametophyte at FG3 with two nuclei separated by a large central vacuole (V). D. A four-nucleate female gametophyte at FG4 with two nuclei at the chalazal pole and the other two nuclei at the micropylar pole. E. An embryo sac at early FG5 stage, with four nuclei at each pole. F. An embryo sac at late FG5 stage. Two polar nuclei locate adjacently to each other. At this stage, cellularization and cell differentiation are completed. There are three antipodal cells (nucleus, AN) at the chalazal pole, two polar nuclei (PN) in the central cell, one egg cell (nucleus, EN), and two synergid cells (nucleus, SN) in the embryo sac. G. An embryo sac at FG6 stage. Two polar nuclei fused to form the central nucleus (CtN). H. A four-celled female gametophyte at FG7. Three antipodal cells were degenerated. Bars=10 µm.

Female gametogenesis in *Arabidopsis* is divided into seven sequential stages [48], namely female gametophyte 1 (FG1) to FG7 (Figure 2). The megaspore mother cell (MMC) undergoes meiosis to form four haploid megaspores. Shortly after the meiosis, three megaspores towards the micropylar pole degenerate manifested as strong autofluorescence under confocal. The chalazal-most megaspore survives to form the functional megaspore that continues to development, entering FG1 phase (Figure 2A). The functional megaspore undergoes one round of mitosis to produce two nuclei, one of which locates at the chalazal pole and the other at the micropylar pole (FG2, Figure 2B). Then, the two nuclei are separated by a central vacuole, entering stage FG3 (Figure 2C). Both nuclei undergo a second round of mitosis to give rise to four nuclei in embryo sac (FG4, Figure 2D). A third round mitosis results in an eight-nucleate embryo sac, with four at each pole (early FG5, Figure 2E). Two polar nuclei migrate along the chalazal–micropylar axis toward each other, and meet at the micropylar half of the embryo sac (late FG5, Figure 2F). Then cellularization takes place simultaneously to produce a seven-celled embryo sac, and the embryo sac enters FG6 upon the two polar nuclei fusion (Figure 2G). Finally, the three antipodal cells degenerate, the remaining four cells, namely the central cell, egg and two synergids, form a female germ unit (FG7, Figure 2H). The female gametophyte is then ready for fertilization.

**Figure 3:**
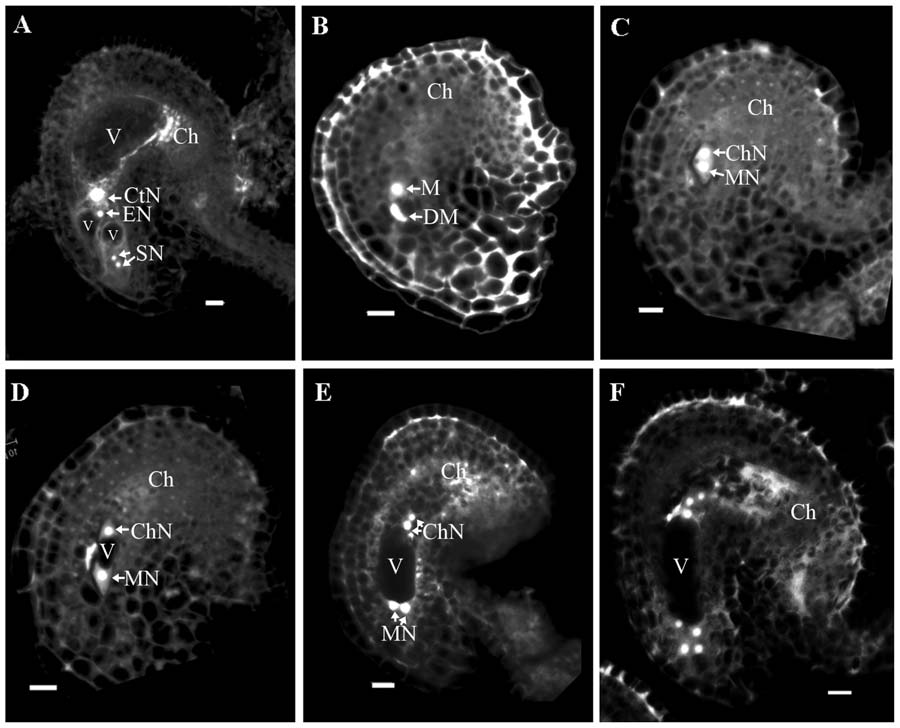
Development of embryo sacs in *gaf1* pistils at flower stage 12c. **A.** An embryo sac at FG7 represents the wild-type ovules in mature *gaf1* pistils. **B–F.** Embryo sacs showed developmental retardation in *gaf1* pistils. After 24 hours emasculated, half of the ovules remained at FG1 (**B**), FG2 (**C**), FG3 (**D**), FG4 (**E**), and early FG5 (**F**). Bars=10 µm.

To investigate developmental defects in the mutant, flowers were emasculated at flower stage 12c [48] and observed 24 hours post emasculation. At this flower stage, most ovules in wild-type pistils were at stage FG6 or FG7. In *gaf1* mutant pistils at the same flower stage, however, only about half of the ovules reached the stage FG6 or FG7 (Figure 3A), and the rest of the ovules were arrested at different stages ranging from FG1 to FG5 (Figure 3B–F). To further check at which stages the ovule development was arrested, a detailed CLSM observation was then carried out in both the mutant and the wild-type pistils. All the buds in the same inflorescence were dissected sequentially, and the developmental stage of each ovule was recorded (Table 2 and Table 3). In wild-type plants, ovules in each pistil predominantly take one or two adjacent developmental stages (Table 2), a phenomenon called developmental synchrony [7], [49]. In contrast to this synchronous development, ovules in *gaf1* pistil span several stages, seven at most in a pistil (Table 3). The mutant embryo sacs stop at any stage, indicating that the synchrony of FG development is seriously impaired.

**Table 2:** Synchrony of Female Gametophyte Development in Wild Type.

Pistil Number	Number of Female Gametophytes at Developmental Stages							
	FG1	FG2	FG3	FG4	FG5	FG6	FG7	Total FGs
1	40	1						41
2	23	25	2					50
3	8	14	16	8				46
4	6	12	33	11				62
5	3	6	16	31	2			58
6			1	9	43			53
7					27	8	10	45
8					3	32	2	37
9					8	13	32	53

**Table 3:** Asynchrony of Female Gametophyte Development in *gaf1*.

Pistil Number	Number of Female Gametophytes at Developmental Stages							
	FG1	FG2	FG3	FG4	FG5	FG6	FG7	Total FGs
1	43	10	2					55
2	14	20	12	13				59
3	15	10	13	14				52
4	8	7	11	13	10			49
5	15	8	11	4	16	3		57
6	14	8	5	8	13	6	4	58
7	2	2	10	11	13	13	3	54
8	1	4	5	12	6	18	3	49
9	4	2	6	6	9	4	28	59

To further investigate whether the retarded mutant embryo sacs have the potential to develop into functional female gametophyte, we performed delayed pollination experiment [7]. Pistils from *gaf1* were emasculated at floral stage 12c, and pollinated with wild-type pollen at 24 h, 48 h, or 72 h post emasculation. The ratio of Kan^R^ progeny was increased significantly in delayed pollination (see Table 4), indicating that a small portion of the mutant ovules have the potential to develop into functional FGs despite the developmental retardation. 

Taken together, *gaf1* mutation affects the normal developmental progression and disrupts the synchrony of female gametophyte development.

**Table 4:** Delayed Pollination Test.

Hours After Emasculation	Progeny	Kan^R^∶ Kan^S^	Std
24	Kan^R^: 5; Kan^S^: 1501	0.0031	0.002241
48	Kan^R^: 36; Kan^S^: 2357	0.0154	0.002721
72	Kan^R^: 23; Kan^S^: 1481	0.0158	0.004448

*gaf1* pistils were pollinated with wild type pollen at 24 h, 48 h, and 72 h after emasculation. At least 2 independent tests were carried out for each group, and the standard deviation is shown in the table.

### The mutation affects slightly pollen competence

**Figure 4:**
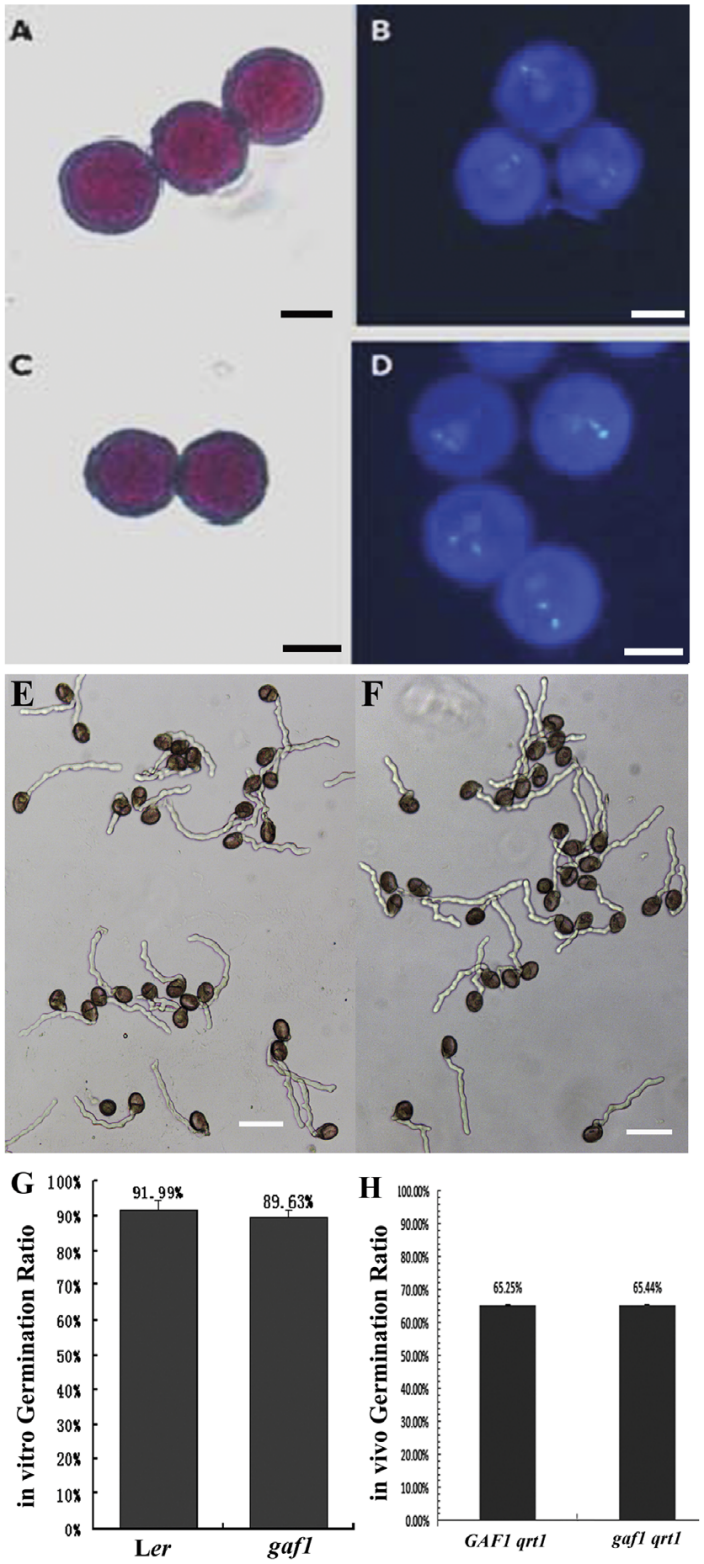
Comparison of pollens from the wild-type and *gaf1* plants. **A** and **C**. Alexander's staining of pollens from the wild-type (**A**) and *gaf1* (**C**) plants. Bars=10 µm. **B** and **D**. DAPI staining of pollens from the wild-type (**B**) and *gaf1* (**D**) plants. Bars=10 µm. **E** and **F**. *in vitro* germination of pollen grains from the wild-type (**E**) and *gaf1* (**F**) plants. Bars=50 µm. **G**. *in vitro* germination ratio of pollen grains from the wild-type (L*er*) and *gaf1* plants. **F**. *in vivo* germination ratio of pollen grains from the *GAF1*/*GAF1 qrt*/*qrt* (*GAF1 qrt*) and *GAF1*/*gaf1 qrt*/*qrt* (*gaf1 qrt*) plants.

Given the low penetration of the mutation via the male gametophyte, experiment was carried out to investigate the developmental or functional defect of the male gametophyte. Mature pollen grains were primarily stained with Alexander's solution [50] and 4′,6-diamino-2-phenylindole (DAPI) staining for their viability and development, respectively. When stained with Alexander's solution, 96.3% of the pollen grains from *gaf1/GAF1* plants showed positive staining (n=1044), compared to 97.7% (n=768) in the wild-type, indicating that *gaf1* did not affect the viability of pollen grains (Figure 4A and 4C). As for DAPI staining, similar to the wild-type pollen grains (98.3%, n=473), most of the grains (98.0%, n=653) from the mutant plants display normal morphology with three nuclei, two small and compact sperm nuclei and one large less condensed vegetative nucleus, suggesting the mitotic progression in *gaf1* pollen development proceeded as the wild-type (Figure 4B and 4D). Furthermore, pollen germination was carried out either *in vitro* or *in vivo*. For the *in vitro* germination test, germination rates for pollen grains from the wild-type and *gaf1* plants are quite similar, and the ratios are 91.99% (n=1582) and 89.63% (n=1855), respectively (Figure 4E–G). The *in vivo* germination were checked with pollen from plants of *GAF1*/*GAF1 qrt*/*qrt* and *gaf1/GAF1 qrt*/*qrt*, the result showed similar germination ratio of pollen grains from plants of the above two genotypes, 65.25% (n=400) for *GAF1*/*GAF1 qrt*/*qrt*, and 65.44% (n=408) for *gaf1/GAF1 qrt*/*qrt* (Figure 4H). 

**Figure 5:**
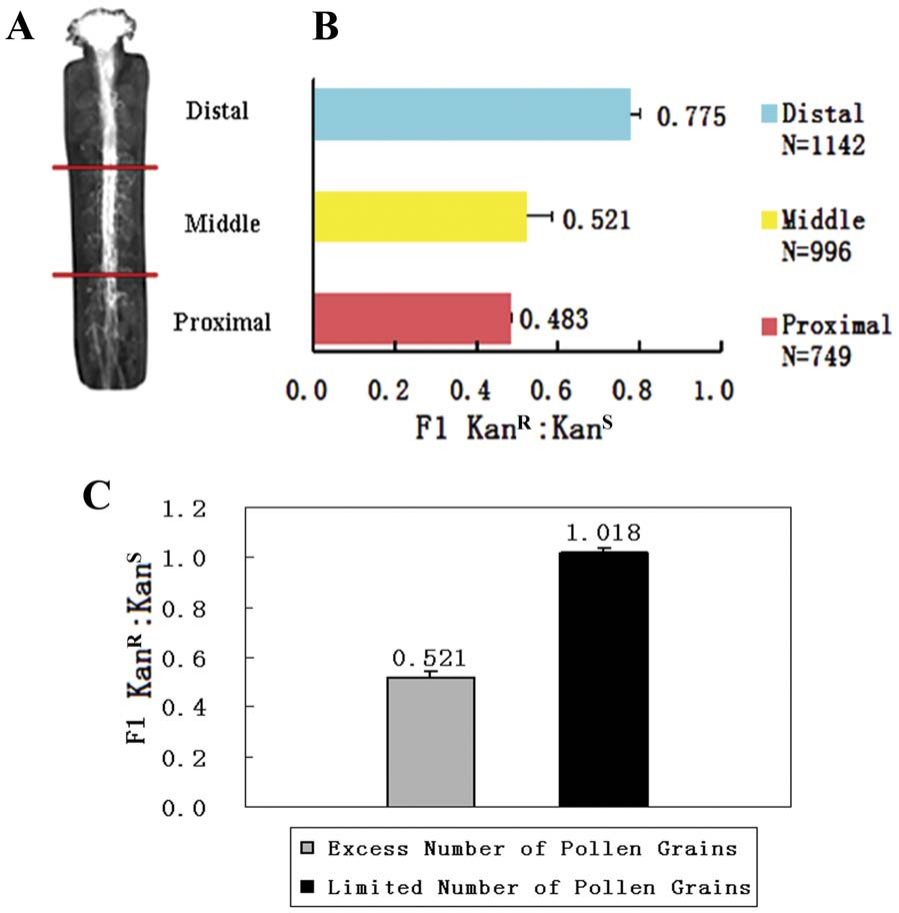
Seed set analysis of *gaf1* mutant plants. **A.** A pistil was divided into three sections according to the distance to stem, namely distal, middle and proximal, from the stigma to stem, respectively. **B.** Kan^R^∶ Kan^S^ ratio of F1 progeny varies in different sections of *gaf1* silique. **C.** Kan^R^∶ Kan^S^ ratio of F1 generation in abundant (1) and limited (2) pollination.

Since *gaf1* pollen grains could germinate *in vivo* normally, it suggests that the defective transmission most likely occurs after germination. The *in vivo* fitness of *gaf1* pollen was studied. Anthers from wild-type flowers at 12c stage were removed, and the emasculated wild-type pistils were pollinated with pollen grains from *gaf1* after 24 h. Every resulting silique from three independent plants was chopped into three equal parts transversely upon maturation, and seeds were collected into three corresponding groups. Seeds of the first group are from that part of silique just next to the stigma, seeds of the second group are from the mid-one-third of the silique, and the seeds from the last part of the silique connecting to the stem were collected as the third group. Curiously, the Kan^R^ segregation ratio of these seedlings varies in these 3 groups. The Kan^R^∶Kan^S^ ratio of the first, second and third group was 0.775 (n=1142), 0.521 (n=996), and 0.483 (n=749), respectively (Figure 5A and 5B). It is obvious that the mutation transmission from pollen grains varies according to the location of the seeds in silique, which means that the ovules close to the stigma have more possibility to be fertilized with mutant *gaf1* pollen, than those ovules further to the stigma, or closer to the stem. It implies that the mutant pollen tubes may be not so competent or elongate so fast as the wild-type tubes, so that the ovules locating further to stigma have less chance to be reached and fertilized by mutant pollen tubes. To elucidate the hypothesis, sparse pollination test was conducted. The pollen grains from *gaf1* plants were dusted on wild-type stigma, and the pollen grains were fewer than 40 for each pistil, to exclude the competition of pollen tubes and make sure that each tube is able to reach an ovule in pistil. Then the seeds were counted for Kan^R^. The data showed that the transmission efficiency through male gametophyte was raised to nearly 100% (Kan^R^∶Kan^S^=1.018, n=1329) when the number of ovules was in excess. We concluded that the mutant pollen tubes can function normally during pollination, however, the fitness of mutant pollen tubes is somehow weaker than those of the wild-type pollens *in vivo*.

### *GAF1* encodes an *Arabidopsis* homologue of yeast RPP1 and human RPP30

**Figure 6:**
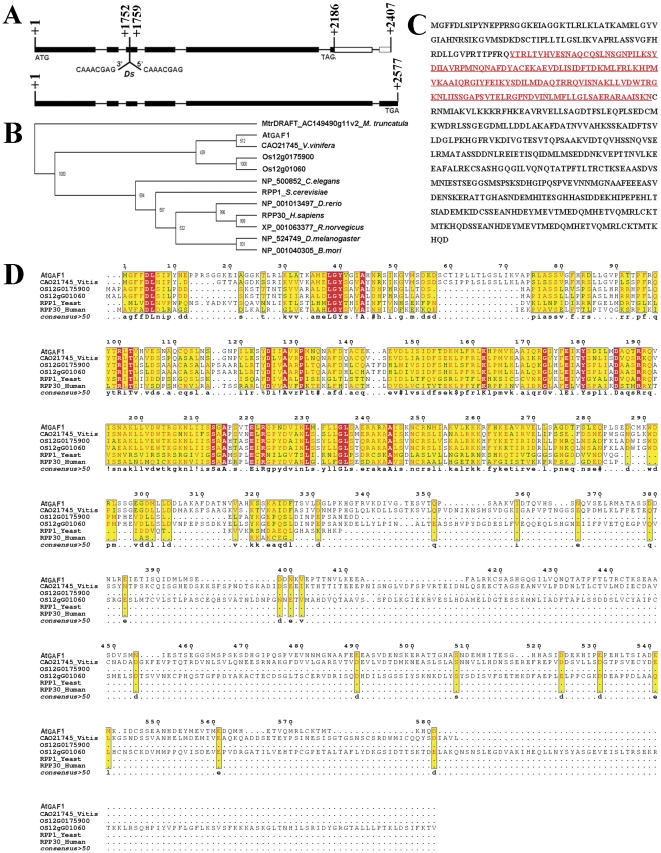
Molecular characterization of *GAF1*. **A.** A scheme showing *GAF1* gene structure and the *Ds* insertion. Black boxes show the ORFs of *GAF1* (*AT5G59980.1*, upper panel) and AT5G56680.2 (lower panel), white boxes show the non-coding region of cDNA and the interval lines depict introns. The numbers of nucleotides are indicated in the figure. **B.** Phylogenetic tree of GAF1 and its homologues from representative organisms. **C.** Amino acid sequence of GAF1. The underlined red sequence is the predicted RPP30 domain. **D.** Alignment of GAF1 and its homologues. Identical amino acids are shown as white letters in red boxes, and similar amino acids are shown as black letters in yellow boxes. GAF1 in *Arabidopsis*; CAO21745, homologue in *Vitis vinifera*; OS12G0175900 and OS12G01060, homologues in *Oryza sativa* (cv *japonica*); RPP1_Yeast, homologue in *Saccharomyces cerevisiae*; RPP30_Human, homologue in *Homo Sapiens*.

In order to identify the *GAF1* gene, *Ds* flanking sequences were cloned with TAIL-PCR [51]. Sequence analysis revealed that the *Ds* element was reversely integrated at position of 1752 bp downstream of ATG start codon, and disturbed the third exon of *At5g59980* (www.arabidopsis.org). The insertion resulted in an 8 bp duplication at the insertion site (Figure 6A). 

The linkage between the *Ds* element and the *gaf1* phenotype was verified. *gaf1* was outcrossed with wild-type plants, and linkage analysis of more than 300 F2 plants showed that, the kanamycin segregation distortion and gametophyte defect are closely linked with the *Ds* insertion. 

Sequence analysis showed that *At5g59980* is a single copy gene in the genome, however, it is predicted that two transcripts are produced from this locus. Transcript *At5g59980.1* is 1986 bp and its genomic sequence (2521 bp) is composed of 7 exons and 6 introns, there are 1746 bp in its open reading frame (ORF), which encodes a peptide of 581 aa. Meanwhile, *At5g59980.2* genomic fragment (2577 bp) contains 6 exons and 5 introns, the cDNA of *At5g59980.2* is 2118 bp, with an ORF of 2118 bp as well, and encoding a peptide of 705 aa. The difference of the two transcripts comes from the 5th exon, which is interrupted with a short intron of 76 bp in *At5g59980.1* and resulting in the shift of the reading frame and an in-frame stop codon in the 6th exon. The predicted At5g59980.1 and At5g59980.2 share identical amino acid sequence in fragment 1–568 aa at the N-terminus (www.arabidopsis.org). According to the prediction, primers were designed and RT-PCR was carried out. Results showed that both of *At5g59980.1* and *At5g59980.2* transcripts were present (data not shown). Therefore, we conducted complementation experiment to identify *GAF1* gene.

First of all, complementation experiment was performed to confirm whether the *Ds* insertion was responsible for *gaf1* phenotype. A 5.1 kb genomic fragment containing 0.6 kb upstream of ATG start codon, 4.5 kb of gene genomic fragments that covering all the exons and introns of both transcripts and sequence downstream the stop codon, was cloned into pCAMBIA1300 and introduced into *gaf1* plants by *Agrobacterium tumefaciens*-mediated infiltration [52]. Seven independent transgenic lines were obtained after selection with hygromycin and kanamycin. Seed sets of these T1 plants were restored to a higher degree (from 73.8% to 90.6% in individual plants), compared with 51.5% in *gaf1* plants. In T2 seeds from these seven lines, the Kan^R^∶Kan^S^ ratio was raised up to 2.62∶1, indicating the 5.3 kb fragment can complement the semisterile phenotype in the *gaf1* background. Furthermore, in 96 transgenic T2 plants, 23 *gaf1/gaf1* homozygous plants were obtained. It confirmed that *Ds* insertion caused the phenotype of the *gaf1* mutant. To further investigate the function of *At5g59980.1* and *At5g59980.2*, we constructed two plasmids, *p_GAF1_*::*GAF1*-*GUS* and *p_GAF1_*::*GAF1*-*EGFP*, in which *GUS* (*β-glucuronidase*) and *EGFP* (*enhanced green fluorescent protein*) genes are inserted before the stop codon TAG of the genomic fragment to yield an in-frame fusion with At5g59980.1, respectively (Figure 6A). The plasmids were used for *Arabidopsis* transformation and the offspring of transgenic plants were scored for Kan^R^, the results showed that both constructs could rescue the phenotype, with the highest ratio of Kan^R^∶Kan^S^ reached to 2.92∶1. It means that *At5g59980.1* is fully functional and sufficient for gametophyte development. *At5g59980.2*, on the other hand, may function redundantly in gametogenesis. So we focused on *At5g59980.1*/*GAF1* in our further study. 

GAF1 is a peptide of 581 aa, with a calculated pI of 5.68 and a molecular weight of 64,080 Da (www.arabidopsis.org). The full length CDS (coding sequence) was amplified and sequenced, it revealed that a conserved RNase P subunit p30 (RPP30) domain was present in GAF1, from the 98^th^ to the 248^th^ aa (http://pfam.sanger.ac.uk/) (Figure 6C). The proteins with RPP30 domain are featured as a shared subunit of RNase P and MRP that are conserved in eukaryotes. Both of RNase P and MRP are consisted of an RNA subunit and one or more protein subunits, they play roles in RNA metabolism [20], [53]. Phylogenetic analysis and blast results show that CAO21745 from *Vitis vinifera*, which also has an RPP30 domain, shares most homology with GAF1, with 64% identity and 81% similarity (Figure 6B, D). Alignment reveals that yeast RPP1 shares 26% identity and 54% similarity with GAF1, with higher homology at the N terminal with RPP30 domain than the C terminal (Figure 6D) among the homologues. RPP1 from yeast has been well studied, which is reported to participate in pre-tRNA and pre-rRNA processing [33].

### *GAF1* is ubiquitously expressed in planta

**Figure 7:**
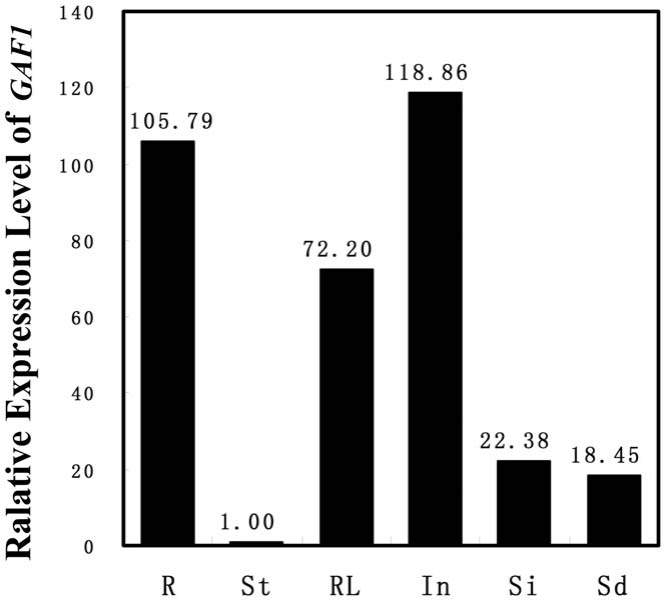
Real-time RT-PCR analysis of *GAF1* expression. Real-time RT-PCR analysis of expression level of *GAF1* in different tissues. Total RNA extracted from root (R), Stem (St), rosette leaf (RL), inflorescence (In), silique (Si), and seedling (Sd) were reverse transcribed and amplified by PCR.

To analyze the expression pattern of *GAF1*, we checked *GAF1* mRNA levels in different tissues with real-time RT-PCR. The results (Figure 7) indicate that *GAF1* mRNA is present in all the tissues tested, and predominantly in inflorescence. 

**Figure 8:**
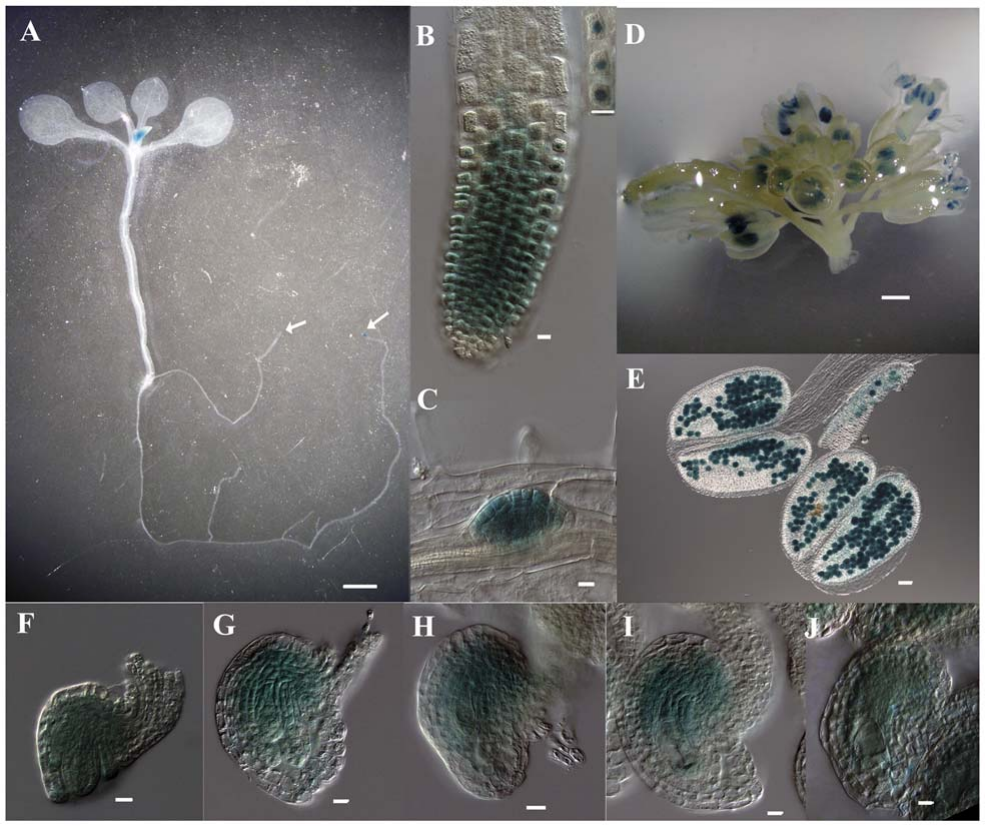
GUS expression during development in transgenic plant p*_GAF1_*:*GAF1*-*GUS*. **A.** A micrograph of a 14d seedling showing GUS signals in root tips (as arrows indicated in A and B) and young leaves. Bar=2 mm. **B.** A micrograph showing GUS signals in root tip. Specific signals were detected in nuclei (inset). Bar=10 µm. **C.** A micrograph showing GUS signals in lateral root primordia. Bar=10 µm. **D.** A micrograph showing GUS signals in inflorescence. Bar=1 mm. **E.** A micrograph showing GUS signals in anthers and pollen grains. Bar=50 µm. **F–J.** Micrographs showing GUS signals in nucellar cells and embryo sacs at different development stages. Bar=10 µm.

*GUS* reporter gene was also used to visualize the expression pattern of *GAF1* in detail. The fusion protein of *GAF1* and *GUS* reporter gene were expressed under the *GAF1* promoter in transgenic plants from construct *p_GAF1_*::*GAF1*-*GUS*. GUS staining results were showed in Figure 8. GUS signal were detected in the young leaves (Figure 8A, arrow), root tip (Figure 8A, arrow and Figure 8B), and lateral primordia (Figure 8C) of 14-day seedlings. In reproductive organs, GUS signal were detected in the inflorescence (Figure 8D), especially in pollen grains (Figure 8E), nucellar cells and embryo sacs of different stages (Figure 8F–J). 

Localization of GAF1 protein was also observed. In the *Arabidopsis* root cells checked (Figure 8B, inset), GUS signal was specifically detected in the nucleus. While in other tissues, GAF1-GUS stain displayed a diffused pattern, suggesting it is also in the cytoplasm (Figure 8). Meanwhile, we also found that GAF1-GFP localized in nucleus and mitochondrion in tobacco leaf cells (data not shown). As the same construction could complement the *gaf1* mutant, the GUS or GFP signal reflects the expression pattern and the subcellular localization of GAF1. This data suggest that GAF1 is localized in both nucleus and mitochondrion.

### GAF1 interacts with *Arabidopsis* homologue of POP5 in yeast

RPP30 is a conserved protein subunit shared by RNase P and MRP, which can be found in all eukaryotic species as well as in Archaea [53]. As *GAF1* encodes the only homologue of RPP30 in *Arabidopsis*, it is likely a protein subunit of RNases P/MRP, and functions in processing pre-tRNA and pre-rRNA. GAF1 homologue RPP1 in yeast is essential for processing pre-tRNA and 35S pre-rRNA, and interacts with POP1, POP3, POP4, POP5, POP6, POP7, POP8, RPR2 (a unique subunit of RNase P), RMP1 and SNM1 (RMP1 and SNM1 are unique subunit of RNase MRP) in pairwise interaction assays [54]. In human, RPP30 was also identified as a scleroderma autoimmune antigen [55], and interacts with RPP14, RPP40, RPP21(homology to yeast RPP2), POP1, RPP38, RPP29 (hPOP4) and RPP30 itself [56], [57]. 

**Figure 9:**
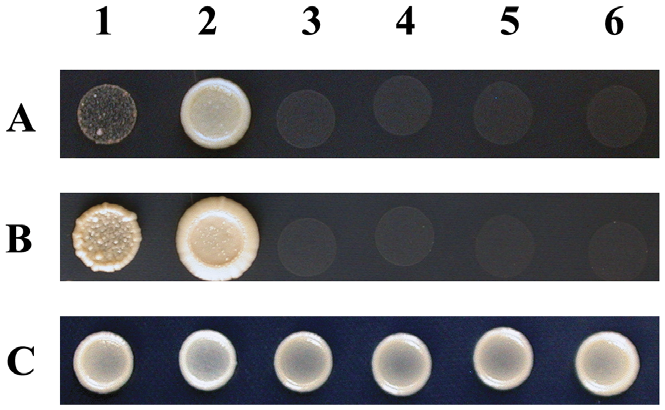
GAF1 interacts with AtPOP5 in yeast cells. Yeast cells were co-transformed with plasmid combinations as indicated. Transformants were growing for 5 days (A) or 12 days (B) at a SD/-Ade-Trp-Leu-His plate, and for 3 days (C) at a SD/-Trp-Leu plate. 1. pGBKT7-AtGAF1+pGADT7-AtPOP5; 2. pGADT7-AtGAF1+pGBKT7-AtPOP5; 3. pGBKT7-AtGAF1+pGADT7; 4. pGADT7-AtGAF1+pGBKT7; 5. pGBKT7-AtPOP5+pGADT7; 6. pGADT7-AtPOP5+ pGBKT7.

As little is known about RNases P/MRP in plants, we searched for homologues of these protein subunits in *Arabidopsis*. Besides GAF1/AtRPP30, five other homologues of the RNases P/MRP complexes were found: At2G47300 (AtPOP1), At2G43190 (AtPOP4), AT1G04635 (AtPOP5), At5G41270 (AtRPR2), and At1G50910 (AtRMP1). In *Arabidopsis*, none of the homologues has been studied. To investigate whether these homologues have similar interaction in *Arabidopsis* as in yeast and human, we checked the protein-protein interaction with yeast two-hybrid system. The full-length CDS of the 6 homologues were cloned and inserted into pGADT7 and pGBKT7 (see Materials and Methods), and then the constructs were co-transformed into yeast cell in pairs. Results showed that GAF1/AtRPP30 can only interact with AtPOP5 (Figure 9), and no interaction were detected between GAF1/AtRPP30 with any other proteins tested. This specific interaction further indicates that GAF1 is a protein subunit of RNases P/MRP. However, interactions between other protein subunits or the unknown protein subunits of *Arabidopsis* RNases P/MRP in plant are not excluded. Our data suggest that RNases P/MRP are critical for female gametogenesis and fitness of male gametophyte in *Arabidopsis*.

## Discussion

Here we showed that the insertion of *Ds* element into *At5g59980* leads to aberrant development of embryo sac and reduced fitness of pollen in *gaf1* mutant. Mutant ovules are defective in the mitotic progression of the embryo sac which leads to developmental arrest, meanwhile, the *gaf1* pollen are less competent than that of the wild-type and cause partial male fertility. These suggest that GAF1 is essential for female gametogenesis and male fitness.

Structural analysis indicated that GAF1 contains a RPP30 domain at its N terminus. It is the only RPP30 homologous protein in *Arabidopsis*. RPP30 is one of the four conserved RNases P/MRP protein subunits in eukaryotes [53]. In yeast, the homologue of RPP30, RPP1, is localized in nucleus [58] and required for pre-tRNA processing and 35S pre-rRNA processing [33]. Meanwhile, RPP1 is essential for yeast viability. Similarly, homozygous *gaf1*/*gaf1* mutant has never been obtained, which emphasizes an essential role of *GAF1*. RPP1 was proved to have interaction with POP1, POP3, POP4 and RPP1 itself in RNase MRP. A recent report indicates that POP5/RPP1 heterodimer exists in RNase MRP [59]. Consistently, GAF1 can interact with AtPOP5 in yeast cells. As well as GAF1, AtPOP5 is the *Arabidopsis* homologue of an eukaryotic conserved RNases P/MRP subunit [19], [20], [60]. All these data suggest that GAF1 is a shared and conserved protein subunit of RNases P/MRP. In yeast and human, both of the GAF1 homologues could interact with more than one protein subunits, and the interacting protein subunits are not exactly the same. This may explain why only the interaction between GAF1 and AtPOP5 was detected. Alternatively, protein-protein interactions are different in different organisms. In addition, the interaction between GAF1 and other proteins might require the presence of a third protein subunit, which might be the conserved ones or specific protein subunit in *Arabidopsis*. Furthermore, obtaining the structure of the two holoenzymes *in vivo* will unveil the truth. Above data indicate that, GAF1, a novel RPP30 homologue in *Arabidopsis*, may function as one of RNases P/MRP subunits through interaction with a proposed subunit AtPOP5.

RNase P and MRP are essential endoribonucleases, they are structurally and functionally related, although they are physically separate complexes [61], [62]. Both of RNase P and MRP are site-specific endoribonuclease. The well-known function of RNase P is the processing of the 5′-end of the pre-tRNA. In addition to the ubiquitous activity of pre-tRNA cleavage, RNase P is also reported to have variety of substrates resembling tRNA, such as 4.5S pre-rRNA [63], [64], operon mRNAs [65], [66], [67], box C/D small nucleolar RNAs. However, only the activity of processing pre-tRNA is the conserved function of RNase P in archaeal, bacterial and eukaryotic kingdoms [68]. In human cells, RNase P is also suggested to play roles in transcription of RNA polymerases I and III, and depletion of RNase P caused reduction of tRNA^Tyr^, tRNA^Met^, 5S rRNA, 7SL RNA and U6 snRNA in Hela cells [29], [62]. Compared with RNase P, RNase MRP is found involved in maturation of 5.8S rRNA in yeast [69], [70], [71], [72]. Via special cleavage of 5′-UTR of *CLB2*, RNase MRP regulates the degradation of *CLB2* mRNA and consequently cell cycle in yeast [27], [31], [32]. As an RPP30 subunit, GAF1 interacts with AtPOP5, another protein unit of RNases P/MRP, and it may possess the ubiquitous function of RNases P/MRP in processing of tRNA and rRNA. However, it is not clear that if plant RNases P/MRP have activity in mRNA degradation, transcription of RNA polymerase, or non-coding RNA generation [73] as in yeast and human cells, since function of plant RNases P/MRP is still poorly understood. Anyway, it is significant that GAF1 may attribute to biogenesis of pre-tRNA and pre-rRNA, just as other components of RNases P/MRP. Since *gaf1* homozygote has never been obtained, we hypothesis that GAF1 may have a global effect on plant development, especially on gametophyte development and competence. Besides the existing gametophytic mutants, such as *swa1*, *swa2*, *swa3*, *gfa1*, *lis*, *maa3*, *ato*, and *nof1*, *gaf1* is the first mutation in plant RNases P/MRP that play a critical role in female gametophyte development and male competence. Our data further endorse the critical roles of RNA biogenesis in gametophyte development and function in plants.

## Materials and Methods

### Plants and growth conditions

*gaf1* and wild-type plants were *Arabidopsis thaliana* Landsberg *erecta* ecotype, and were grown in air-conditioned room under a 16-h light/8-h dark cycle. The mutant was isolated from a *Ds* gene trap population as reported previously [74]. Seeds were sterilized in 20% bleach for 10 min, and rinsed 5 times with sterilized water, then germinated on MS medium. Antibiotic was added as required: 50 mg/L kanamycin for kanamycin selection; 20 mg/L hygromycin for hygromycin selection. Plant transformation was performed by *Agrobacterium tumefaciens*–mediated infiltration [52].

### Phenotype analysis

Plant pollination and confocal observation of the embryo sacs of *gaf1* and wild-type plants were performed as described previously [7]. Inflorescences or emasculated pistils were fixed in 4% glutaraldehyde in 12.5 mM cacodylate (pH6.9), and the plant tissues were dehydrated through a conventional ethanol series. Pistils were dissected and mounted in immersion oil before observation. The samples were observed with Zeiss LSM510 META laser scanning microscope (Zeiss, Jena, Germany) with a 488-nm argon laser and an LP 530 filter.

Alexander's and DAPI staining assays were conducted as described before [46], [50]. *In vitro* pollen germination was carried out according to the methods in Jiang and colleagues [75]. For *in vivo* germination, a *qrt/qrt* background was used. A few tetrad pollen grains from *gaf1/GAF1 qrt/qrt* plants were pollinated on the wild-type stigma 24 h after emasculated at flower stage 12c, then fixed and stained with aniline blue [76] 12 h post pollination. Pollen and pollen tubes were observed with a Zeiss Axioskop II microscope equipped with a Zeiss AxioCam MRc5 camera.

### Molecular cloning and complementation

The sequence information comes from The *Arabidopsis* Information Resource (TAIR) (http://www.arabidopsis.org). A 5049 bp genomic fragment, including 575 bp promoter region and 2288 bp 3′ UTR of *At5g59980*, was amplified with primer 286-gF-*Kpn* and 286-gR-*Sac* (the primers used in this research are listed in table 5). The fragment was subcloned into *pCAMBIA1300* at *Kpn* I and *Sac* I sites. Positive construct was introduced into *gaf1* mutant plants by Agrobacterium-mediated infiltration [52]. 

The *GUS* coding sequence was amplified from *pWM101* [77] with primers GUSF-*Kpn* and GUSRV-*Pst*. The *GUS* fragment was then subcloned into *pCAMBIA1300* at *Kpn* I and *Pst* I sites to produce *p1300*-*GUS*. A genomic fragment containing 575 bp upstream of the ATG start codon and the genomic sequence of *At5g59980.1* and 2288 bp downstream of the TGA stop codon were amplified with primer combinations 286promoterF-*Hin*d/286gnscR-*Hin*d and 286-3utrF-*Sac*/286-gR-*Sac*, respectively. Two fragments were then subcloned into *p1300-GUS* at *Hin*d III and *Sac* I, respectively, to generate construct *p_GAF1_*::*GAF1*-*GUS*. The *GUS* CDS in *p_GAF1_*::*GAF1*-*GUS* was replaced by *EGFP*, and *p_GAF1_*::*GAF1*-*EGFP* was constructed. All constructs were confirmed by sequencing.

**Table 5:** Primers used in this research.

Name of Primer	Sequence of Primer (from 5′- to -3′)
286-gF-*Kpn*	CGGGGTACCAAGCCTTTTCCTCCCTTTCCACGAC
286-gR-*Sac*	ACGGAGCTCTTCTGTCACATCATATTTGGGTCTAAGG
GUSF-*Kpn*	GGGGTACCATGTTACGTCCTGTAGAAAC
GUSRV-*Pst*	AACTGCAGTCATTGTTTGCCTCCCTGCT
286promoterF-*Hin*d	ACGTAAGCTTAAGCCTTTTCCTCCCTTTCCACGAC
286gnscR-*Hin*d	ACGTAAGCTTGTCTTGATGCTTCGTCATGGTCTTG
286-3utrF-*Sac*	ACGGAGCTCAGTCCGATGAGACAAAGCTCGAGGA
286-gR-*Sac*	ACGGAGCTCTTCTGTCACATCATATTTGGGTCTAAGG
At5g59980cDNA-F	ATGGGATTCTTCGATCTTAGC
At5g59980.1cDNA-MR	CGCATCTGGACTGTTTCATG
At5g59980.2cDNA-R	TCAATGTTTTCTTTTTTTACTGATC
At5g59980.2cDNA-R2	TTGAACCTTGATTGAAGTAG
286-Q1F	GAACACCTCACATCCATTGC
286-Q1R	TCATGCATCTGGTCTTCCAT
POP1-F-*Nde*	GGGAATTCCATATGATGGCTACTACTGCGAATGGAAAC
POP1-R-*Nde*	GGGAATTCCATATGATGGCTACTACTGCGAATGGAAAC
POP1-R-*Nde*	GGGAATTCCATATGTTAAAAGCAGTGGACATCACAATAGTC
*POP4-F-EcoR*	*CCGGAATTCATGGGTACAGAGACGGTTGTGCATG*
POP4-R-*Eco*R	GCCGGAATTCTTAACGTTGAACAATATTGTCTCTTG
POP5-F-*Eco*R	CCGGAATTCATGGTGGGATTTAAGAACAGATACATG
POP5-R-*Eco*R	CCGGAATTCTCAGTTTTCCAATAGTTTGATCTTCTC
RPR2-F-*Eco*R	CCGGAATTCATGGGCAAACGAGGACCGAAGAAG
RPR2-R-*Eco*R	GCCGGAATTCTTACAAGAGAAAAGGTATTTTAAAGTTTC
RPP30-F-*Nde*	GGGAATTCCATATGATGGGATTCTTCGATCTTAGCATTC
RPP30-R-*Nde*	GGGAATTCCATATGCTAGTCTTGATGCTTCGTCATGGTC
RMP1-F-*Eco*R	CCGGAATTCATGGATGATACACAAGTTAAAGC
RMP1-R-*Eco*R	CCGGAATTCCTAGAACAAACTGTCTTTCTGTG

### Phylogenetic analysis and sequence alignment

The RPP30 homologous protein sequences of different organisms were obtained from National Center for Biotechnology Information (NCBI) using Blastp (http://www.ncbi.nih.nlm.gov). The amino acid sequences of the conserved RPP30 domain of the homologous proteins were used to generate a neighbor joining phylogenetic tree with Phylip3.68 software. Multiple protein sequence alignments were performed by ClustalW at NPS@Clustal (http://www.ebi.ac.uk/Tools/clustalw2/index.html).

### RT-PCR analysis

Total RNA was isolated from wild-type Landsberg *erecta* with TRIzol (Invitrogen, USA), and digested with RQ1 DNase I (Promega, USA). cDNA was synthesized by SuperScriptII (Invitrogen, USA). Specific primers At5g59980cDNA-F/At5g59980.1cDNA-MR were used to amplify *At5g59980.1* cDNA, At5g59980cDNA-F/At5g59980.2cDNA-R, or At5g59980cDNA-F/At5g59980.2cDNA-R2 were used for amplification of *At5g59980.2* cDNA. 

Real-time PCR was performed using SYBR Green Master mix (Applied Biosystems) and ABI 7900 sequence detection system. For *GAF1* amplification, primer 286-Q1F and primer 286-Q1R were used, and amplification of *Actin2/8* mRNA [78] was used as an internal control.

### GUS staining

GUS staining of plants transformed with *p_GAF1_*::*GAF1*-*GUS* was performed and observed as described previously [13], [76]. Tissues were incubated in GUS staining solution containing 1 mg/mL 5-bromo-4-chloro-3-indoxyl-beta-D-glucuronide cyclohexylammonium salt (X-Gluc), 1 mM potassium ferrocyanide, 1 mM potassium ferricyanide, 10 mM EDTA, 0.1% Triton X-100, and 100 mg/mL chloramphenicol in 50 mM sodium phosphate buffer, pH 7.0) for 3 to 5 days at 37°C after an initial 30 min vacuum. The stained samples were cleared in 20% lactic acid/20% glycerol solution, and observed by a Zeiss Axioskop II microscope.

### Yeast two hybrid assay

The sequences of *Arabidopsis* homologues of RNases P/MRP protein subunits were obtained from NCBI and TAIR (as mentioned above). CDS of AtPOP1, AtPOP4, AtPOP5, AtRPR2, AtRPP30/GAF1, and AtRMP1 were amplified with following primer pairs: POP1-F-*Nde* and POP1-R-*Nde*; POP4-F-*Eco*R and POP4-R-*Eco*R; POP5-F-*Eco*R and POP5-R-*Eco*R; RPR2-F-*Eco*R and RPR2-R-*Eco*R; RPP30-F-*Nde* and RPP30-R-*Nde*; RMP1-F-*Eco*R and RMP1-R-*Eco*R. CDS fragments were cloned into both pGADT7 and pGBKT7 at *Eco*R I or *Nde* I. Constructs were confirm with sequencing and transformed into AH109 as described in Yeast Protocol Handbook (Clonetech, USA). The transformed cells were adjusted to OD_600_=0.3 and grown on SD/-Trp-Leu plates for 3 days and on SD/-Trp-Leu-His-Ade plates for 5 or 12 days at 28°C.
